# Synthesis, crystal structure and Hirshfeld analysis of a new crystalline modification of the radical ion salt octa­methyl­ene­tetra­thia­fulvalenium triiodide (OMTTF)I_3_

**DOI:** 10.1107/S2056989018013907

**Published:** 2018-10-09

**Authors:** Adriano Bof de Oliveira, Johannes Beck, Jörg Daniels

**Affiliations:** a Departamento de Química, Universidade Federal de Sergipe, Av. Marechal Rondon s/n, 49100-000 São Cristóvão-SE, Brazil; b Institut für Anorganische Chemie, Rheinische Friedrich-Wilhelms-Universität Bonn, Gerhard-Domagk-Strasse 1, D-53121 Bonn, Germany

**Keywords:** crystal structure, synthetic metals, tetra­thia­fuvalenium radical salt, tetra­thia­fuvalenium polyiodides

## Abstract

The reaction between 4,5,6,7-tetra­hydro-2-(4,5,6,7-tetra­hydro-1,3-benzodi­thiol-2-yl­idene)-1,3-benzodi­thiole (common name: 4,4′,5,5′,6,6′,7,7′-octa­hydro­dibenzo­tetra­thia­fulvalene, OMTTF) and an excess of iodine in tetra­hydro­furan (THF) yielded the respective radical organic polyiodide salt, C_14_H_16_S_4_^+^·I_3_^−^. The asymmetric unit contains one and a half formula unit of both the cation and the anion, with the half-ions completed through inversion symmetry. The (OMTTF ^+^) positive charge can be assigned by the bond distances and the planar structure of the C_2_S_2_C=CS_2_C_2_ central fragment. In the crystal, trimers of triiodide anions are connected through secondary inter­molecular I⋯I inter­actions into almost linear I_9_^3−^ polyanions. The non-centrosymmetric OMTTF radical cations are linked by S⋯S inter­actions into centrosymmetric dimers, while the centrosymmetric OMTTF cations remain as discrete units. The (OMTTF ^+^) radical cations and the triiodide anions are linked by weak C—H⋯I and C—H⋯S inter­actions into a three-dimensional network. This work reports the fourth crystalline modification of the C_14_H_16_S_4_  ^+^·I_3_^−^ salt. The three previous modifications were obtained from a mixture of aceto­nitrile and toluene [Konarev *et al.* (2005 ▸). *Synth. Met.* **151**, 231–238].

## Abstract 2

A new crystalline phase of the radical ion salt octa­methyl­ene­tetra­thia­fulvalenium triiodide is reported. The synthesis in THF, the crystal structure and the Hirshfeld surface analysis are described and the new modification, designated as δ-(OMTTF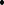 ^+^)(I_3_^−^), is compared with the three previously known forms.

## Chemical context   

Tetra­thia­fulvalenes (TTF) belong to one of the most important and promising classes of sulfur-containing compounds in materials chemistry, with an emphasis on their electrical conductivity and magnetic properties. As far as we know, the first report about a sulfur-containing heterocycle with the C_2_S_2_C=CS_2_C_2_ central fragment, dibenzo­tetra­thia­fulvalene, can be traced back to the early twentieth century (Hurtley & Smiles, 1926 ▸). In the 1970′s and 1980′s, the focus of TTF research changed rapidly from heterocyclic synthetic chemistry to materials chemistry as a result of the wide range of applications in electric and magnetic devices (Fanghänel *et al.*, 1983 ▸; Hünig *et al.*, 1973*a* ▸,*b* ▸; Richter *et al.*, 1984 ▸; Schukat & Fanghänel, 1979 ▸, 1982 ▸; Schukat *et al.*, 1979 ▸, 1976 ▸, 1981 ▸, 1982*a* ▸,*b* ▸). TTF and its derivatives can be synthesized and manipulated as common organic substances, but they can show properties normally associated with metals or semi-metals. The tetra­thia­fulvalene–tetra­cyano­quinodi­methane compound (TTF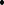 ^δ+^)_*m*_(TCNQ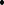 ^δ-^)_*n*_ is considered to be the first *synthetic metal* because of its high electrical and metallic conductivity down to 53 K. At lower temperatures, a Peierls distortion under pair formation occurs, accompanied by transition to semiconducting behaviour (Wudl *et al.*, 1970 ▸). A further breakthrough within the field of TTF chemistry came with the synthesis of bis­(ethyl­endi­thio)­tetra­thia­fulvalene (BEDT-TTF), which opened a completely new area for materials science: superconductivity in mol­ecular systems. (BEDT-TTF)_2_(I_3_) is an example of a polyiodide superconductor radical salt at 7 K. In its crystal structure, the (BEDT-TTF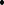 ^δ+^) units are stacked along the [110] direction with short inter­molecular C⋯C contacts. Short inter-stack S⋯S inter­actions along [100] complete the organic substructure, a layer parallel to (001). As a result of the partially positive charge over the columns of (BEDT-TTF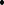 ^δ+^) and the short contacts, electrical conductivity is directed along this axis. The I_3_^−^ units form the inorganic subcell, layers parallel to (001), which enables the crystal packing and ensures electrostactic neutrality (Madsen *et al.*, 1999 ▸).

A key point within TTF chemistry is the oxidation of the related sulfur-containing mol­ecule to a radical cation with integral or partial charge. The fully oxidized TTF derivatives show salt structures and structure-correlated magnetic properties, while partially oxidized ones show charge-transfer-like behaviour. The radical electron and the positive charge are mainly located over the central fragment of the mol­ecule, the C_2_S_2_C=CS_2_C_2_ unit, being stabilized by the delocalization of electron density from the sulfur atoms to the central C
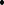
—^+^C fragment and by the two five-membered rings, which become aromatic in the course of the oxidation. In addition, the electron-density delocalization increases the symmetry of the central unit, from *C*_2v_ to *D*_2h_, which contributes to the cation stability. A huge number of different anions can be used with TTF-derivative cations, from simple halide ions to coordination compounds, resulting in a great diversity of mol­ecular arrangements, supra­molecular structures and physicochemical properties (Saito & Yoshida, 2007 ▸). TTF and its derivatives can also be chemically oxidized with metal salts. For example, the reaction of OMTTF with an excess of CuBr_2_ yields the salt (OMTTF^2+^)_2_[Cu^I^_2_Cu^II^_2_Br_10_^4−^] (Beck & Oliveira, 2009 ▸). Here the central C1^+^—^+^C2 fragment loses two electrons and the resulting C—C bond length is 1.449 (12) Å, a value similar to single bond lengths. Accordingly, rotation between the rings connected by the two central carbon atoms becomes possible and the angle between the mean planes of the two five-membered rings is 15.34°.

Another key consideration in TTF chemistry is the magnetic properties of the compounds. (TTF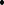 ^+^) radical deriv­atives are paramagnetic because of the unpaired electron. Neutral TTF and dicationic (TTF^2+^) derivatives are diamagnetic and a paramagnetic susceptibility will be only possible with the use of paramagnetic anions. For the (OMTTF^2+^)_2_[Cu^I^_2_Cu^II^_2_Br_10_^4−^] salt compound, the anion shows an inter­esting structure formed by four metal centers with mixed oxidation states and connected tetra­hedral coordination polyhedra, building a unique anionic complex showing anti­ferromagnetic coupling between the Cu^II^ centres (Beck & Oliveira, 2009 ▸).
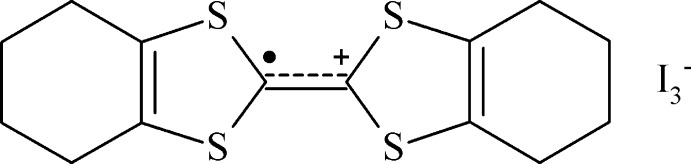


As part of our research on TTF organic radical chemistry, we report herein the synthesis, crystal structure and Hirshfeld analysis of a new crystalline modification of the octa­methyl­entetra­thia­fulvalenium triiodide (OMTTF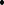 ^+^)(I_3_^−^) compound. Three crystalline modifications of this TTF derivative salt structure have already been reported (Konarev *et al.*, 2005 ▸).

## Structural commentary   

**Figure 1:**
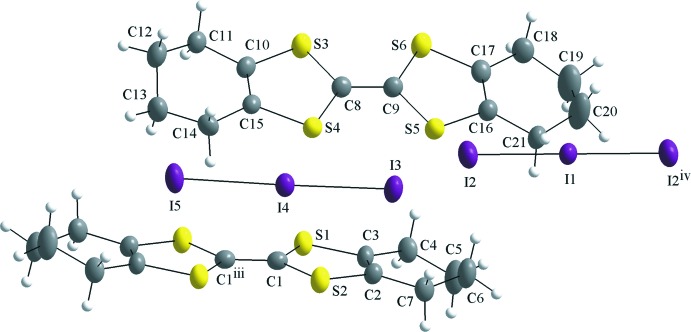
The mol­ecular structure of the title compound, δ-(OMTTF)I_3_, showing the atom labelling and displacement ellipsoids drawn at the 40% probability level. Symmetry codes: (iii) −*x* + 1, −*y* + 1, −*z*; (iv) −*x* + 2, −*y*, −*z* + 1.

There are one and a half mol­ecules of both the cation and the anion in the asymmetric unit (Fig. 1 ▸) of octa­methyl­entetra­thia­fulvalenium triiodide (OMTTF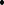 ^+^)(I_3_^−^), (C_14_H_16_S_4_)I_3_, both half-ions being completed by inversion symmetry. The two OMTTF units are fully oxidized to the +1 charge and the electron density is delocalized over the C_2_S_2_C
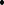
—^+^CS_2_C_2_ fragment, as implied by their inter­mediate bond lengths. The central C—C distance is consistent with increased single-bond character due to the loss of one electron. For neutral OMTTF, the central C—C bond length is 1.326 (4) Å (Zaman *et al.*, 1999 ▸), while in this work the values are 1.405 (7) Å for C1—C1^iii^ [symmetry code: (iii) −*x* + 1, −*y* + 1, −*z*] and 1.383 (5) Å for C8—C9. At the same time, the S—C bond distances are consistent with increased double-bond character, since the sulfur atoms polarize the electron density to the central C—C bond to stabilize the positive charge and the two five-membered rings become aromatic. For neutral OMTTF, the S—C bond distance is 1.759 (3) Å. For the (OMTTF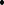 ^+^) mol­ecule in this work, the S—C bond distances are S1—C1 = 1.719 (3), S2—C1 = 1.715 (4), S3—C8 = 1.711 (4), S4—C8 = 1.720 (4), S5—C9 = 1.727 (4) and S6—C9 = 1.724 (4) Å. This change in the bond character is a key feature in distinguishing between neutral and cationic TTF derivatives and is well known in the literature (Zaman *et al.*, 1999 ▸). The non-planarity of the six-membered rings is shown by the maximum deviation from the mean plane of the non-H atoms for the (OMTTF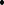 ^+^) mol­ecules, 0.307 (4) Å for C5 and 0.415 (4) Å for C13. The central fragments of the mol­ecules are nearly planar, with a maximum deviation from the mean plane of the non-H atoms of the C8–C10/C15–C17/S3–S6 fragment of 0.0790 (18) Å for S6 and a maximum deviation from the C1–C3/S1/S2/C1^iii^–C3^iii^/S1^iii^/S2^iii^ fragment of 0.0221 (11) Å for S2. The r.m.s deviations of their atoms from these mean planes are 0.0504 and 0.016 Å, respectively. In the triiodide anion I2–I1–I2^iv^ [symmetry code: (iv) −*x* + 2, −*y*, −*z* + 1], the two I—I bonds are identical; in the second anion I3–I4–I5, the two I—I bonds differ only by 0.06 Å. Thus, polarization of the anions by the cations can be regarded as negligible (Fig. 1 ▸).

## Supra­molecular features   

**Figure 2:**
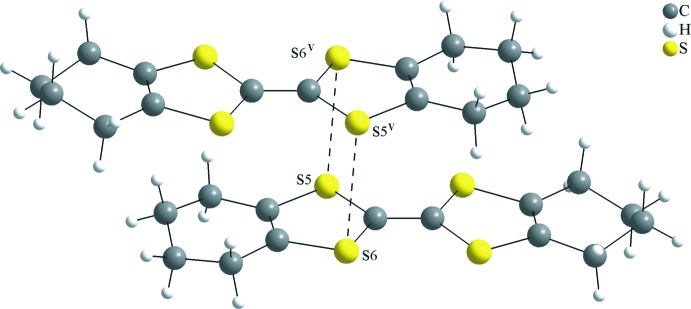
Representation of the centrosymmetric dimer (OMTTF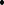 ^+^)_2_^2+^ of the title compound. The S⋯S inter­molecular inter­actions are drawn as dashed lines. Symmetry code: (v) −*x* + 1, −*y* + 1, −*z* + 1.

**Figure 3:**
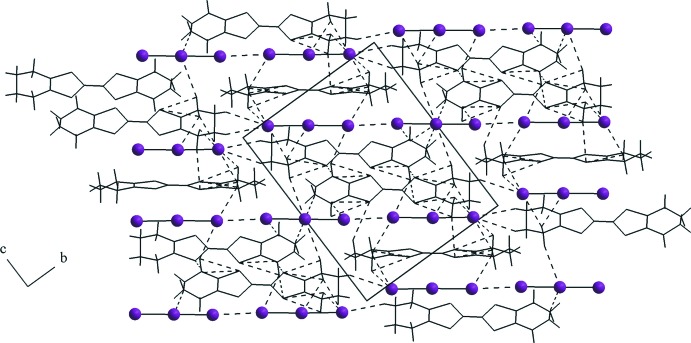
Section of the crystal structure of the title compound, δ-(OMTTF)I_3_, showing the three-dimensional hydrogen-bonded network built up by H⋯I inter­molecular inter­actions (shown as dashed lines). The I_3_^−^ units are linked by I⋯I inter­molecular inter­actions, also drawn as dashed lines, forming one-dimensional chains of nine I atoms. The figure is simplified for clarity.

(OMTTF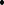 ^+^)(I_3_^−^) is an organic radical compound with a salt structure. The cations and anions alternate in the crystal structure and no π–π stacking, organic radical columns or an organic conductor subcell is observed, as *e.g.* for the (BEDT-TTF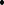 ^δ+^)_*m*_(I_3_^−^)_*n*_ superconductor (Madsen *et al.*, 1999 ▸). In the crystal structure of the title compound, pairs of non-centrosymmetric (OMTTF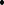 ^+^) units are connected through S⋯S inter­molecular inter­actions into inversion dimers. The sum of the van der Waals radii for S⋯S contacts is 3.6 Å (Bondi, 1964 ▸; Rowland & Taylor, 1996 ▸) and the distance for the S5⋯S6^v^ inter­action amounts to 3.4841 (16) Å (Fig. 2 ▸) [symmetry code: (v) −*x* + 1, −*y* + 1, −*z* + 1]. Meanwhile, the centrosymmetric OMTTF cations remain as discrete units, with no short inter­molecular contacts. The triiodide anions show a close inter­molecular I⋯I distance of 3.5934 (5) Å, much shorter than the sum of the van der Waals radii, 3.96 Å (Bondi, 1964 ▸; Rowland & Taylor, 1996 ▸), and forming an almost linear polyiodide anion I_9_^3−^ aligned with the [021] direction. Finally, the (OMTTF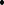 ^+^) entities and the I_3_^−^ anions are linked to each other by C—H⋯I and C—H⋯S hydrogen bonds, forming a three-dimensional network (Fig. 3 ▸ and Table 1 ▸). Additionally, there are very weak inter­molecular contacts with H⋯I distances from 3.21 to 3.38 Å and with a H⋯S distance of 3.00 Å, which are longer the sum of the van der Waals radii for the respective atoms but are relevant for the crystal cohesion (Fig. 3 ▸ and *Hirshfeld analysis* section).

## Hirshfeld surface analysis   

**Figure 4:**
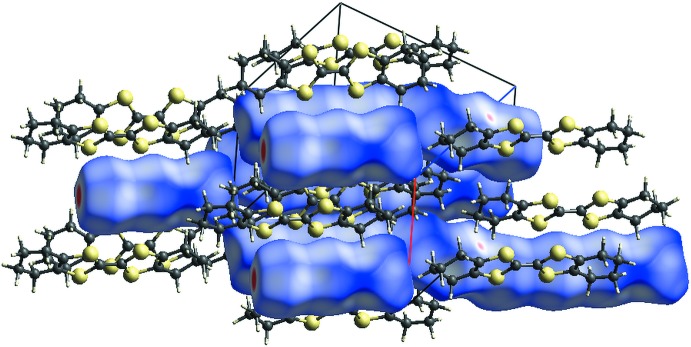
Section of the crystal structure of the title compound, δ-(OMTTF)I_3_. The Hirshfeld surface representation (*d*_norm_) is drawn for the I atoms, while the (OMTTF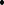 ^+^) entities are drawn using a ‘ball-and-stick’ model for clarity. The surface regions with strongest inter­molecular inter­actions are shown in magenta, the (I_3_^−^)⋯(I_3_^−^) inter­actions for example. The surface regions with weak inter­actions, *e.g.* the H⋯I contacts, are pale magenta in colour. The strongest and the frequently observed inter­molecular inter­actions (in %) are not necessarily are the same. The *a* axis is drawn in red and the *c* axis is drawn in blue.

**Figure 5:**
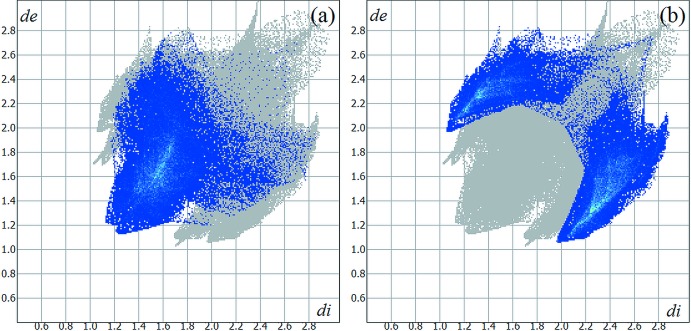
Hirshfeld surface fingerprint plot for the title compound showing (*a*) the H⋯H and (*b*) the H⋯I contacts in detail (cyan dots). The contribution of the these inter­molecular inter­actions to the crystal packing amounts to 31.40 and 34.60%, respectively.

The Hirshfeld surface graphical representation (Hirshfeld, 1977 ▸) of the polyiodide oligomers in a section of the crystal structure indicates, in magenta, the locations of the I⋯I inter­molecular contacts, while the (OMTTF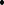 ^+^) units are represented using a ‘ball-and-stick’ model for clarity (Fig. 4 ▸). The (I_3_^−^)⋯(I_3_^−^) contacts are the most striking features in the Hirshfeld surface figure, but the most important contribution to the crystal cohesion (in %) comes from the H⋯H and H⋯I inter­molecular inter­actions, amounting to 31.40 and 34.60% of the surface contacts, respectively. The selected contributions to the crystal packing are shown as two-dimensional Hirshfeld surface fingerprint plots with cyan dots (Wolff *et al.*, 2012 ▸). The *d*_e_ (*y* axis) and *d*_i_ (*x* axis) values are the distances to the closest external and inter­nal atoms (values in Å) from a given point on the Hirshfeld surface (Fig. 5 ▸ *a* for H⋯H and 5*b* for H⋯I).

## Database survey   

**Figure 6:**
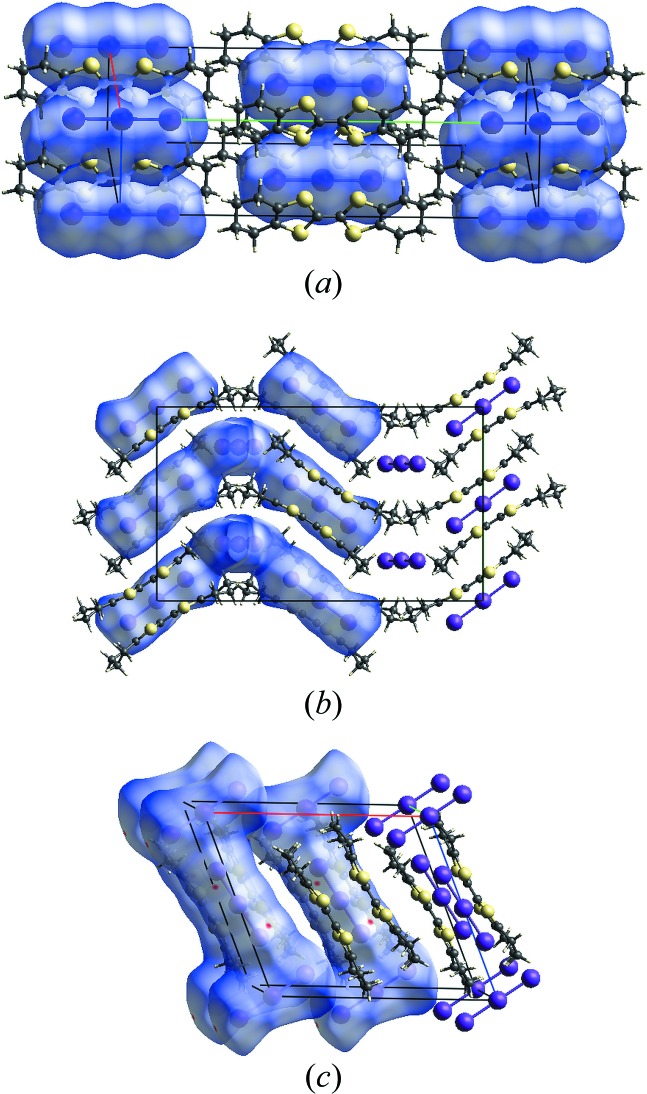
Sections of the crystal structures of the (*a*) α, (*b*) β and (*c*) γ polymorphs of (OMTTF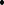 ^+^)(I_3_^−^). For details about the structures, please see: the *Database survey* section of this work and Konarev *et al.* (2005 ▸). For clarity, the I_3_^−^ units are drawn in different formats: either as Hirshfeld surface representations (*d*_norm_) or using ‘ball-and-stick’ models. The *a* axis is drawn in red, the *b* axis is drawn in green and the *c* axis in blue. The cell in (*b*) is viewed along the *a* axis.

To the best of our knowledge and using database tools such as SciFinder (Chemical Abstracts Service, 2018 ▸), three different crystalline polymorphs of (OMTTF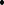 ^+^)(I_3_^−^) have been reported (Konarev *et al.*, 2005 ▸), now designated as α [monoclinic, *C*2/*m*, *a* = 7.7696 (8), *b* = 25.2965 (15) and *c* = 5.7335 (6) Å], β [ortho­rhom­bic, *C*222_1_, *a* = 7.7696 (8), *b* = 25.2965 (15) and *c* = 5.7335 (6)] and γ [also monoclinic, *C*2/*m*, *a* = 16.670 (1), *b* = 9.182 (1) and *c* = 14.426 (1) Å] (Fig. 6 ▸ *a* for the α-, Fig. 6*b* for the β and Fig. 6*c* for the γ form). All three crystalline modifications are obtained by the same synthetic route and from a mixture of aceto­nitrile and toluene as crystallization solvent. All three polymorphs show a salt structure, and the new δ polymorph fits into the series. There are, however, distinct differences in the inter­actions between the radical cations in the respective structures. In the β (Fig. 6 ▸ *b*) and the γ (Fig. 6 ▸ *c*) modifications, the OMTTF radical ions form π dimers (OMTTF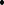 ^+^)_2_^2+^. The mol­ecules are arranged in a parallel fashion. Two four-center two-electron bonds between the S atoms are present with typical S⋯S distances around 3.3 Å. The inter­molecular bonds between the central parts of the two mol­ecules cause steric repulsion in the peripheral parts. As a consequence, the mol­ecules are no longer planar but achieve a typical bent shape. For the δ modification, a centrosymmetric dimer (OMTTF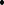 ^+^)_2_^2+^ is observed for the radical cations without a mol­ecular inversion centre. Unlike in the β and γ forms, in the δ form the (OMTTF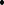 ^+^) units of the dimer are not face-to-face, but offset (Fig. 2 ▸).

**Figure 7:**
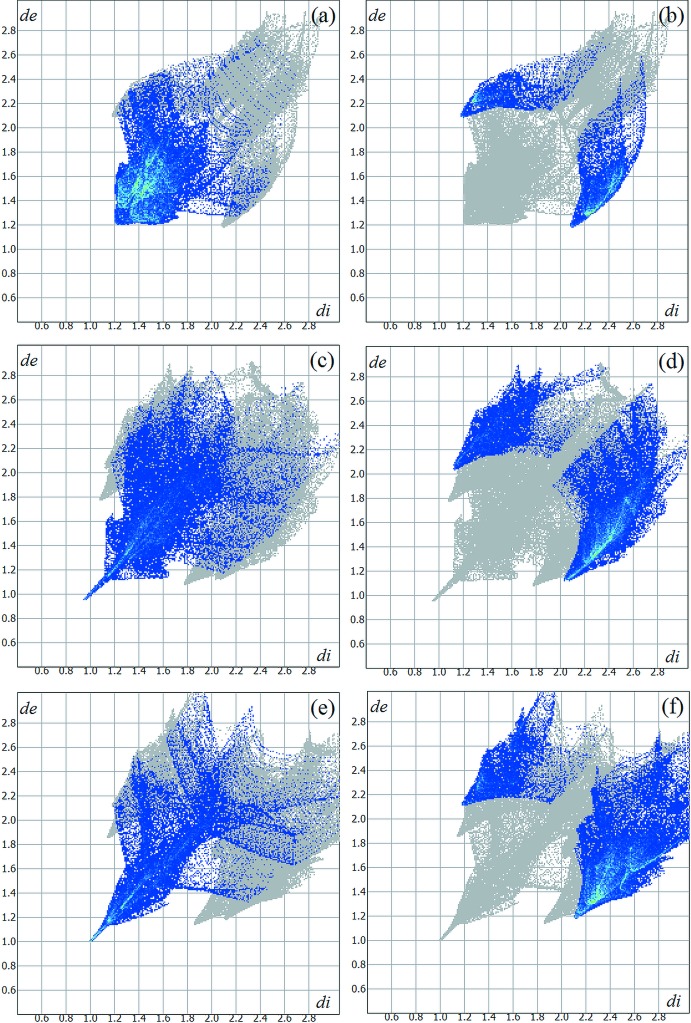
Two-dimensional Hirshfeld surface fingerprint plots for the α, β and γ (OMTTF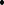 ^+^)(I_3_^−^) crystalline modifications. The H⋯H and H⋯I inter­molecular contacts are drawn as cyan dots. The contributions of these inter­actions for the crystal packing of the α form amount to (*a*) 43.30% and (*b*) 17.40%, respectively. For the β form the values amount to (*c*) 29.20% and (*d*) 33.90% and, finally, for the γ form the values are (*e*) 23.00% and (*f*) 39.70%.

In the α and the δ forms, concerning the centrosymmetric radical cations only, the (OMTTF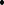 ^+^) units are mainly isolated from each other. Only weak inter­molecular inter­actions with S⋯S distances longer than the sum of the van der Waals radii of two sulfur atoms are observed. This is in line with the almost undistorted planarity of the entire mol­ecule. For the three modifications, some I_3_^−^ units were drawn as Hirshfeld surfaces (Hirshfeld, 1977 ▸) and some others as ‘ball-and-stick’ models for clarity. The selected contributions to the crystal packing are shown as two-dimensional Hirshfeld surface fingerprint plots with cyan dots (Wolff *et al.*, 2012 ▸). The analysis of the complete asymmetric units of the three crystalline modifications suggests that the contribution of the H⋯H and H⋯I contacts for the crystal cohesion are 43.30% and 17.40% for the α modification (Fig. 7 ▸ *a* and 7*b*). For the β modification, the values for the H⋯H and H⋯I contacts amount to 29.20% and 33.90% (Fig. 7 ▸ *c* and 7*d*). Finally, for the γ modification the values for the selected contacts amount to 23.00% and 39.70% (Fig. 7 ▸ *e* and 7*f*). The H⋯H and H⋯I contacts were selected for comparison and analysis of the four crystalline modifications because they are the most frequent (in percentage terms) for all structures, but still show clear differences between the polymorphs.

## Synthesis and crystallization   

All starting materials are commercially available and were used without further purification. In order to obtain fully oxidized TTF radical cations, an excess of I_2_ was employed. In a typical experiment, OMTTF (3.00 × 10 ^−4^ mol) and iodine (1.20 × 10 ^−3^ mol) were separately dissolved in anhydrous tetra­hydro­furane (40 mL). The solutions were added separately and simultaneously to each tubing of a U-shaped Schlenk flask previously evacuated and filled with argon. As the U shape was divided into two compartments by a level 3 porosity frit, the diffusion of the two solutions was slow. After some weeks, black crystals suitable for X-ray diffraction were obtained. The OMTTF radical cation triiodide is air-sensitive in solution, but stable for years in the solid state.

## Refinement   

Crystal data, data collection and structure refinement details are summarized in Table 2 ▸. Hydrogen atoms were positioned with idealized geometry and refined isotropically using a riding model, with *U*_iso_(H) = 1.2 *U*_eq_(C), and with C—H = 0.97 Å. The possibility of disorder was verified with a new refinement, but rejected. Although the shapes of the displacement ellipsoid for C20, C21 and C5 are different from those of nearby atoms, these peripheral *sp* ^3^ atoms have more freedom to move and no clear splitting was suggested by the data.

## Supplementary Material

Crystal structure: contains datablock(s) I. DOI: 10.1107/S2056989018013907/fy2131sup1.cif

Structure factors: contains datablock(s) I. DOI: 10.1107/S2056989018013907/fy2131Isup2.hkl

Supporting information file. DOI: 10.1107/S2056989018013907/fy2131Isup3.cml

CCDC reference: 1871276

Additional supporting information:  crystallographic information; 3D view; checkCIF report

## Figures and Tables

**Table 1:** Hydrogen-bond geometry (Å, °)

*D*—H⋯*A*	*D*—H	H⋯*A*	*D*⋯*A*	*D*—H⋯*A*
C18—H23⋯S3^i^	0.97	2.80	3.669 (4)	150
C21—H17⋯I1^ii^	0.97	3.06	3.763 (4)	131

Symmetry codes: (i) 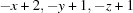; (ii) 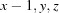.

**Table 2:** Experimental details

Crystal data	
Chemical formula	C_14_H_16_S_4_^+^·I_3_^−^
*M*_r_	693.21
Crystal system, space group	Triclinic, *P* 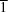
Temperature (K)	293
*a*, *b*, *c* (Å)	8.4334 (1), 12.1857 (2), 14.9874 (3)
α, β, γ (°)	90.063 (1), 94.279 (1), 104.063 (1)
*V* (Å^3^)	1489.59 (4)
*Z*	3
Radiation type	Mo *K*α
μ (mm^−1^)	5.13
Crystal size (mm)	0.21 × 0.20 × 0.03

Data collection	
Diffractometer	Nonius KappaCCD
Absorption correction	Analytical (Alcock, 1970 ▸)
*T*_min_, *T*_max_	0.375, 0.859
No. of measured, independent and observed [*I* > 2σ(*I*)] reflections	30936, 6630, 4525
*R*_int_	0.064
(sin θ/λ)_max_ (Å^−1^)	0.651

Refinement	
*R*[*F* ^2^ > 2σ(*F* ^2^)], *wR*(*F* ^2^), *S*	0.031, 0.066, 1.01
No. of reflections	6630
No. of parameters	287
H-atom treatment	H-atom parameters constrained
Δρ_max_, Δρ_min_ (e Å^−3^)	0.60, −0.58

Computer programs: *COLLECT* (Nonius, 1998 ▸), *HKL*, *DENZO* and *SCALEPACK* (Otwinowski & Minor, 1997 ▸), *SHELXT2014* (Sheldrick, 2015*a* ▸), *SHELXL2016* (Sheldrick, 2015*b* ▸), *WinGX* (Farrugia, 2012 ▸), *DIAMOND* (Brandenburg, 2006 ▸), *publCIF* (Westrip, 2010 ▸) and *enCIFer* (Allen *et al.*, 2004 ▸).

## supplementary crystallographic information

### Crystal data

**Table:**

C_14_H_16_S_4_^+^·I_3_^−^	*Z* = 3
*M**_r_* = 693.21	*F*(000) = 969
Triclinic, *P*1	*D*_x_ = 2.318 Mg m^−^^3^
*a* = 8.4334 (1) Å	Mo *K*α radiation, λ = 0.71073 Å
*b* = 12.1857 (2) Å	Cell parameters from 6128 reflections
*c* = 14.9874 (3) Å	θ = 2.9–27.5°
α = 90.063 (1)°	µ = 5.13 mm^−^^1^
β = 94.279 (1)°	*T* = 293 K
γ = 104.063 (1)°	Plate, black
*V* = 1489.59 (4) Å^3^	0.21 × 0.20 × 0.03 mm

### Data collection

**Table:**

Nonius KappaCCD diffractometer	6630 independent reflections
Radiation source: fine-focus sealed tube, Enraf Nonius FR590	4525 reflections with *I* > 2σ(*I*)
Detector resolution: 9 pixels mm^-1^	*R*_int_ = 0.064
CCD rotation images, thick slices scans	θ_max_ = 27.6°, θ_min_ = 2.9°
Absorption correction: analytical (Alcock, 1970)	*h* = −10→10
*T*_min_ = 0.375, *T*_max_ = 0.859	*k* = −15→15
30936 measured reflections	*l* = −19→19

### Refinement

**Table:**

Refinement on *F*^2^	Secondary atom site location: difference Fourier map
Least-squares matrix: full	Hydrogen site location: inferred from neighbouring sites
*R*[*F*^2^ > 2σ(*F*^2^)] = 0.031	H-atom parameters constrained
*wR*(*F*^2^) = 0.066	*w* = 1/[σ^2^(*F*_o_^2^) + (0.0266*P*)^2^] where *P* = (*F*_o_^2^ + 2*F*_c_^2^)/3
*S* = 1.01	(Δ/σ)_max_ = 0.001
6630 reflections	Δρ_max_ = 0.60 e Å^−^^3^
287 parameters	Δρ_min_ = −0.58 e Å^−^^3^
0 restraints	Extinction correction: SHELXL2016 (Sheldrick, 2015b), Fc^*^=kFc[1+0.001xFc^2^λ^3^/sin(2θ)]^-1/4^
Primary atom site location: structure-invariant direct methods	Extinction coefficient: 0.00314 (13)

### Special details

**Table:**

Geometry. All esds (except the esd in the dihedral angle between two l.s. planes) are estimated using the full covariance matrix. The cell esds are taken into account individually in the estimation of esds in distances, angles and torsion angles; correlations between esds in cell parameters are only used when they are defined by crystal symmetry. An approximate (isotropic) treatment of cell esds is used for estimating esds involving l.s. planes.

### Fractional atomic coordinates and isotropic or equivalent isotropic displacement parameters (Å^2^)

**Table:**

	*x*	*y*	*z*	*U*_iso_*/*U*_eq_	
C1	0.4855 (4)	0.4532 (3)	0.0283 (2)	0.0362 (9)	
C2	0.3544 (5)	0.2803 (3)	0.1170 (2)	0.0376 (9)	
C3	0.5172 (5)	0.2928 (3)	0.1320 (2)	0.0380 (9)	
C4	0.5919 (5)	0.2134 (3)	0.1886 (3)	0.0452 (11)	
H7	0.681449	0.195920	0.158918	0.054*	
H8	0.636151	0.249881	0.245726	0.054*	
C5	0.4663 (6)	0.1060 (4)	0.2038 (3)	0.0699 (15)	
H5	0.454486	0.057149	0.151362	0.084*	
H6	0.507530	0.067726	0.253869	0.084*	
C6	0.3023 (5)	0.1197 (4)	0.2222 (3)	0.0639 (13)	
H3	0.310645	0.158159	0.279685	0.077*	
H4	0.229222	0.045277	0.226698	0.077*	
C7	0.2274 (5)	0.1851 (3)	0.1522 (3)	0.0467 (11)	
H1	0.174160	0.134295	0.103131	0.056*	
H2	0.144579	0.215407	0.178135	0.056*	
S1	0.64287 (12)	0.40535 (9)	0.08132 (7)	0.0430 (3)	
S2	0.29183 (12)	0.37907 (9)	0.04903 (7)	0.0459 (3)	
C8	0.6525 (4)	0.5857 (3)	0.3377 (2)	0.0363 (9)	
C9	0.6279 (4)	0.4954 (3)	0.3950 (2)	0.0380 (9)	
C10	0.7741 (4)	0.7704 (3)	0.2585 (2)	0.0359 (9)	
C11	0.8902 (5)	0.8796 (3)	0.2380 (3)	0.0417 (10)	
H15	0.982120	0.864480	0.209144	0.050*	
H16	0.932298	0.921952	0.293088	0.050*	
C12	0.8012 (6)	0.9493 (4)	0.1762 (3)	0.0591 (12)	
H13	0.733418	0.983985	0.211080	0.071*	
H14	0.881546	1.009379	0.150601	0.071*	
C13	0.6958 (6)	0.8785 (4)	0.1021 (3)	0.0597 (13)	
H11	0.763997	0.844999	0.066617	0.072*	
H12	0.646144	0.926795	0.063457	0.072*	
C14	0.5609 (5)	0.7848 (3)	0.1366 (2)	0.0456 (10)	
H9	0.474640	0.816605	0.156809	0.055*	
H10	0.513735	0.730336	0.088775	0.055*	
C15	0.6317 (5)	0.7260 (3)	0.2137 (2)	0.0352 (9)	
C16	0.5244 (5)	0.3034 (3)	0.4685 (3)	0.0403 (10)	
C17	0.6676 (5)	0.3521 (3)	0.5147 (3)	0.0411 (10)	
C18	0.7419 (5)	0.2956 (4)	0.5897 (3)	0.0490 (11)	
H23	0.856148	0.301230	0.580341	0.059*	
H24	0.738052	0.334997	0.645504	0.059*	
C19	0.6560 (6)	0.1743 (4)	0.5972 (4)	0.0866 (18)	
H21	0.711564	0.129966	0.562472	0.104*	
H22	0.669841	0.153589	0.659273	0.104*	
C20	0.4869 (6)	0.1409 (5)	0.5699 (4)	0.096 (2)	
H19	0.427943	0.160412	0.618302	0.115*	
H20	0.457257	0.059102	0.563713	0.115*	
C21	0.4236 (5)	0.1871 (3)	0.4858 (3)	0.0511 (11)	
H17	0.310604	0.189950	0.490762	0.061*	
H18	0.426010	0.137188	0.435721	0.061*	
S3	0.82518 (12)	0.69438 (9)	0.34950 (7)	0.0466 (3)	
S4	0.51549 (12)	0.59778 (9)	0.24947 (7)	0.0421 (3)	
S5	0.45793 (13)	0.38249 (9)	0.38374 (7)	0.0438 (3)	
S6	0.76624 (13)	0.48567 (9)	0.48358 (8)	0.0486 (3)	
I1	1.000000	0.000000	0.500000	0.04237 (11)	
I2	0.96057 (4)	0.18959 (3)	0.38707 (2)	0.05908 (11)	
I3	0.02295 (4)	0.43954 (3)	0.25421 (2)	0.05695 (10)	
I4	0.08720 (3)	0.63328 (2)	0.13886 (2)	0.04368 (9)	
I5	0.15401 (4)	0.83474 (3)	0.02504 (2)	0.05716 (11)	

### Atomic displacement parameters (Å^2^)

**Table:**

	*U*^11^	*U*^22^	*U*^33^	*U*^12^	*U*^13^	*U*^23^
C1	0.035 (2)	0.035 (2)	0.037 (2)	0.0074 (18)	−0.0009 (17)	−0.0009 (17)
C2	0.037 (3)	0.036 (2)	0.039 (2)	0.0082 (19)	0.0024 (18)	0.0057 (18)
C3	0.044 (3)	0.032 (2)	0.039 (2)	0.0088 (18)	0.0067 (18)	0.0079 (18)
C4	0.040 (3)	0.049 (3)	0.051 (3)	0.020 (2)	0.0036 (19)	0.016 (2)
C5	0.063 (3)	0.062 (3)	0.092 (4)	0.026 (3)	0.014 (3)	0.037 (3)
C6	0.060 (3)	0.058 (3)	0.073 (3)	0.012 (3)	0.005 (2)	0.029 (3)
C7	0.038 (3)	0.052 (3)	0.047 (2)	0.006 (2)	0.0036 (19)	0.012 (2)
S1	0.0330 (6)	0.0437 (6)	0.0507 (6)	0.0065 (5)	0.0018 (5)	0.0150 (5)
S2	0.0352 (6)	0.0459 (7)	0.0568 (7)	0.0106 (5)	0.0021 (5)	0.0178 (5)
C8	0.029 (2)	0.040 (2)	0.042 (2)	0.0100 (18)	0.0037 (17)	0.0039 (18)
C9	0.032 (2)	0.038 (2)	0.043 (2)	0.0073 (18)	0.0011 (18)	0.0026 (19)
C10	0.029 (2)	0.035 (2)	0.044 (2)	0.0071 (18)	0.0069 (18)	0.0050 (18)
C11	0.038 (2)	0.039 (2)	0.045 (2)	0.0023 (19)	0.0045 (18)	0.0049 (19)
C12	0.059 (3)	0.047 (3)	0.068 (3)	0.008 (2)	0.003 (2)	0.015 (2)
C13	0.068 (3)	0.054 (3)	0.052 (3)	0.006 (3)	−0.004 (2)	0.015 (2)
C14	0.047 (3)	0.050 (3)	0.038 (2)	0.011 (2)	−0.0065 (19)	0.005 (2)
C15	0.038 (2)	0.033 (2)	0.034 (2)	0.0066 (18)	0.0073 (17)	0.0006 (17)
C16	0.039 (2)	0.038 (2)	0.043 (2)	0.0081 (19)	0.0017 (19)	0.0055 (19)
C17	0.036 (2)	0.043 (2)	0.046 (2)	0.0126 (19)	0.0064 (19)	0.0052 (19)
C18	0.038 (2)	0.054 (3)	0.055 (3)	0.013 (2)	−0.004 (2)	0.011 (2)
C19	0.073 (4)	0.077 (4)	0.106 (5)	0.017 (3)	−0.010 (3)	0.043 (3)
C20	0.079 (4)	0.073 (4)	0.115 (5)	−0.007 (3)	−0.031 (3)	0.050 (3)
C21	0.055 (3)	0.040 (3)	0.051 (3)	0.001 (2)	0.000 (2)	0.010 (2)
S3	0.0322 (6)	0.0483 (7)	0.0548 (7)	0.0036 (5)	−0.0046 (5)	0.0145 (5)
S4	0.0373 (6)	0.0416 (6)	0.0420 (6)	0.0005 (5)	−0.0019 (5)	0.0044 (5)
S5	0.0422 (6)	0.0401 (6)	0.0437 (6)	0.0025 (5)	−0.0064 (5)	0.0061 (5)
S6	0.0328 (6)	0.0491 (7)	0.0582 (7)	0.0012 (5)	−0.0045 (5)	0.0139 (5)
I1	0.0429 (2)	0.0382 (2)	0.0419 (2)	0.00195 (17)	0.00331 (17)	0.00827 (17)
I2	0.0742 (2)	0.0530 (2)	0.05357 (19)	0.02108 (16)	0.00849 (15)	0.02068 (15)
I3	0.0598 (2)	0.04395 (18)	0.0629 (2)	0.00554 (15)	0.00173 (15)	0.01986 (15)
I4	0.04059 (18)	0.04315 (17)	0.04686 (17)	0.00971 (13)	0.00192 (12)	0.01106 (12)
I5	0.0543 (2)	0.0586 (2)	0.0610 (2)	0.01688 (15)	0.00964 (15)	0.02880 (15)

### Geometric parameters (Å, º)

**Table:**

C1—C1^i^	1.405 (7)	C12—C13	1.498 (6)
C1—S2	1.715 (4)	C12—H13	0.9700
C1—S1	1.719 (3)	C12—H14	0.9700
C2—C3	1.346 (5)	C13—C14	1.525 (6)
C2—C7	1.501 (5)	C13—H11	0.9700
C2—S2	1.731 (4)	C13—H12	0.9700
C3—C4	1.508 (5)	C14—C15	1.519 (5)
C3—S1	1.731 (4)	C14—H9	0.9700
C4—C5	1.500 (6)	C14—H10	0.9700
C4—H7	0.9700	C15—S4	1.740 (4)
C4—H8	0.9700	C16—C17	1.347 (5)
C5—C6	1.479 (6)	C16—C21	1.498 (5)
C5—H5	0.9700	C16—S5	1.737 (4)
C5—H6	0.9700	C17—C18	1.496 (5)
C6—C7	1.510 (5)	C17—S6	1.721 (4)
C6—H3	0.9700	C18—C19	1.489 (6)
C6—H4	0.9700	C18—H23	0.9700
C7—H1	0.9700	C18—H24	0.9700
C7—H2	0.9700	C19—C20	1.413 (7)
C8—C9	1.383 (5)	C19—H21	0.9700
C8—S3	1.711 (4)	C19—H22	0.9700
C8—S4	1.720 (4)	C20—C21	1.496 (5)
C9—S6	1.724 (4)	C20—H19	0.9700
C9—S5	1.727 (4)	C20—H20	0.9700
C10—C15	1.322 (5)	C21—H17	0.9700
C10—C11	1.496 (5)	C21—H18	0.9700
C10—S3	1.736 (4)	I1—I2	2.9344 (3)
C11—C12	1.533 (5)	I2—I1^ii^	2.9344 (3)
C11—H15	0.9700	I3—I4	2.8954 (4)
C11—H16	0.9700	I4—I5	2.9555 (4)
			
C1^i^—C1—S2	122.6 (4)	C11—C12—H14	109.2
C1^i^—C1—S1	122.0 (4)	H13—C12—H14	107.9
S2—C1—S1	115.4 (2)	C12—C13—C14	112.6 (4)
C3—C2—C7	124.3 (3)	C12—C13—H11	109.1
C3—C2—S2	116.5 (3)	C14—C13—H11	109.1
C7—C2—S2	119.2 (3)	C12—C13—H12	109.1
C2—C3—C4	123.2 (4)	C14—C13—H12	109.1
C2—C3—S1	117.0 (3)	H11—C13—H12	107.8
C4—C3—S1	119.8 (3)	C15—C14—C13	109.7 (3)
C5—C4—C3	110.8 (3)	C15—C14—H9	109.7
C5—C4—H7	109.5	C13—C14—H9	109.7
C3—C4—H7	109.5	C15—C14—H10	109.7
C5—C4—H8	109.5	C13—C14—H10	109.7
C3—C4—H8	109.5	H9—C14—H10	108.2
H7—C4—H8	108.1	C10—C15—C14	123.3 (4)
C6—C5—C4	115.6 (4)	C10—C15—S4	117.8 (3)
C6—C5—H5	108.4	C14—C15—S4	118.8 (3)
C4—C5—H5	108.4	C17—C16—C21	124.0 (4)
C6—C5—H6	108.4	C17—C16—S5	116.4 (3)
C4—C5—H6	108.4	C21—C16—S5	119.6 (3)
H5—C5—H6	107.4	C16—C17—C18	123.2 (4)
C5—C6—C7	114.2 (4)	C16—C17—S6	116.9 (3)
C5—C6—H3	108.7	C18—C17—S6	119.9 (3)
C7—C6—H3	108.7	C19—C18—C17	112.5 (4)
C5—C6—H4	108.7	C19—C18—H23	109.1
C7—C6—H4	108.7	C17—C18—H23	109.1
H3—C6—H4	107.6	C19—C18—H24	109.1
C2—C7—C6	111.6 (3)	C17—C18—H24	109.1
C2—C7—H1	109.3	H23—C18—H24	107.8
C6—C7—H1	109.3	C20—C19—C18	118.2 (4)
C2—C7—H2	109.3	C20—C19—H21	107.8
C6—C7—H2	109.3	C18—C19—H21	107.8
H1—C7—H2	108.0	C20—C19—H22	107.8
C1—S1—C3	95.36 (18)	C18—C19—H22	107.8
C1—S2—C2	95.70 (17)	H21—C19—H22	107.1
C9—C8—S3	121.7 (3)	C19—C20—C21	119.5 (5)
C9—C8—S4	123.4 (3)	C19—C20—H19	107.4
S3—C8—S4	115.0 (2)	C21—C20—H19	107.4
C8—C9—S6	122.6 (3)	C19—C20—H20	107.4
C8—C9—S5	123.1 (3)	C21—C20—H20	107.4
S6—C9—S5	114.3 (2)	H19—C20—H20	107.0
C15—C10—C11	125.5 (3)	C20—C21—C16	111.1 (4)
C15—C10—S3	115.9 (3)	C20—C21—H17	109.4
C11—C10—S3	118.5 (3)	C16—C21—H17	109.4
C10—C11—C12	109.7 (3)	C20—C21—H18	109.4
C10—C11—H15	109.7	C16—C21—H18	109.4
C12—C11—H15	109.7	H17—C21—H18	108.0
C10—C11—H16	109.7	C8—S3—C10	96.23 (17)
C12—C11—H16	109.7	C8—S4—C15	95.03 (18)
H15—C11—H16	108.2	C9—S5—C16	95.82 (18)
C13—C12—C11	112.2 (4)	C17—S6—C9	96.25 (19)
C13—C12—H13	109.2	I2—I1—I2^ii^	180.0
C11—C12—H13	109.2	I3—I4—I5	178.593 (13)
C13—C12—H14	109.2		
			
C7—C2—C3—C4	−1.8 (6)	S3—C10—C15—S4	−0.9 (4)
S2—C2—C3—C4	−179.2 (3)	C13—C14—C15—C10	17.4 (5)
C7—C2—C3—S1	177.3 (3)	C13—C14—C15—S4	−166.5 (3)
S2—C2—C3—S1	−0.1 (4)	C21—C16—C17—C18	−0.7 (7)
C2—C3—C4—C5	15.3 (6)	S5—C16—C17—C18	179.9 (3)
S1—C3—C4—C5	−163.8 (3)	C21—C16—C17—S6	179.1 (3)
C3—C4—C5—C6	−41.0 (6)	S5—C16—C17—S6	−0.2 (4)
C4—C5—C6—C7	54.4 (6)	C16—C17—C18—C19	9.7 (6)
C3—C2—C7—C6	12.3 (6)	S6—C17—C18—C19	−170.2 (3)
S2—C2—C7—C6	−170.3 (3)	C17—C18—C19—C20	−29.8 (7)
C5—C6—C7—C2	−37.3 (6)	C18—C19—C20—C21	42.4 (9)
C1^i^—C1—S1—C3	−178.2 (4)	C19—C20—C21—C16	−30.3 (8)
S2—C1—S1—C3	1.7 (3)	C17—C16—C21—C20	9.7 (7)
C2—C3—S1—C1	−1.0 (3)	S5—C16—C21—C20	−170.9 (3)
C4—C3—S1—C1	178.1 (3)	C9—C8—S3—C10	179.3 (3)
C1^i^—C1—S2—C2	178.1 (4)	S4—C8—S3—C10	−0.2 (2)
S1—C1—S2—C2	−1.8 (3)	C15—C10—S3—C8	0.7 (3)
C3—C2—S2—C1	1.2 (3)	C11—C10—S3—C8	179.7 (3)
C7—C2—S2—C1	−176.4 (3)	C9—C8—S4—C15	−179.7 (3)
S3—C8—C9—S6	1.0 (5)	S3—C8—S4—C15	−0.1 (2)
S4—C8—C9—S6	−179.5 (2)	C10—C15—S4—C8	0.6 (3)
S3—C8—C9—S5	−178.3 (2)	C14—C15—S4—C8	−175.7 (3)
S4—C8—C9—S5	1.2 (5)	C8—C9—S5—C16	174.4 (3)
C15—C10—C11—C12	16.4 (6)	S6—C9—S5—C16	−5.0 (3)
S3—C10—C11—C12	−162.5 (3)	C17—C16—S5—C9	3.2 (3)
C10—C11—C12—C13	−43.9 (5)	C21—C16—S5—C9	−176.2 (3)
C11—C12—C13—C14	61.3 (5)	C16—C17—S6—C9	−2.8 (4)
C12—C13—C14—C15	−45.3 (5)	C18—C17—S6—C9	177.0 (3)
C11—C10—C15—C14	−3.7 (6)	C8—C9—S6—C17	−174.5 (3)
S3—C10—C15—C14	175.2 (3)	S5—C9—S6—C17	4.9 (3)
C11—C10—C15—S4	−179.8 (3)		

Symmetry codes: (i) −*x*+1, −*y*+1, −*z*; (ii) −*x*+2, −*y*, −*z*+1.

### Hydrogen-bond geometry (Å, º)

**Table:**

*D*—H···*A*	*D*—H	H···*A*	*D*···*A*	*D*—H···*A*
C18—H23···S3^iii^	0.97	2.80	3.669 (4)	150
C21—H17···I1^iv^	0.97	3.06	3.763 (4)	131

Symmetry codes: (iii) −*x*+2, −*y*+1, −*z*+1; (iv) *x*−1, *y*, *z*.
